# Cesarean section and childhood infections: Causality for concern?

**DOI:** 10.1371/journal.pmed.1003457

**Published:** 2020-11-19

**Authors:** Gordon C. S. Smith

**Affiliations:** Department of Obstetrics & Gynaecology, University of Cambridge, Cambridge, United Kingdom

## Abstract

In this Perspective, Gordon Smith discusses the findings of Miller et al, and the balance of risks and benefits associated with different modes of delivery.

Human birth where the infant survives without passing through the mother’s genital tract has only been recorded in the past few thousand years, with accounts of cesarean delivery in antiquity. Cases where the mother also survives the surgery are more recent still, with the first authenticated description of successful cesarean delivery in 1610 [1]. The development and implementation of safe cesarean delivery in the late 19th and 20th centuries were transformative for maternal and infant survival in high-income countries. Globally, the availability of cesarean delivery is now 1 of the most important elements of emergency obstetric care to reduce rates of maternal and perinatal death [2].

In the past century, however, cesarean delivery has transformed from occasional lifesaving surgery to the most commonly performed laparotomy, accounting for 1 in 14 of all surgical procedures worldwide in 2012 [3]. The indications for its use, once lethal and absolute, have been supplemented with many relative indications. Maternal preference for the method in the absence of any medical indication is increasing [4]. Given that there is widespread international variation in the proportion of female obstetricians choosing to have elective cesarean deliveries themselves [5], variation in practice reflects differences in interpretation of the same evidence base.

Deciding on cesarean section depends on understanding its risks and benefits, and a huge volume of data exists to inform women. Interpretation of elements of the evidence base may not be simple, such as associations with the actual mode of delivery [6], which, unlike the intended mode of delivery, cannot be known when making a decision. While there is extensive information on the short-term associations, the long-term effects on the both the mother and child are less well documented. This week in *PLOS Medicine*, a cohort study including 7.1 million live births from 4 countries, reported by Jessica Miller and colleagues, provides useful new evidence and raises the possibility that cesarean delivery may be associated with the risk of later childhood infectious disease [7]. The authors performed a pooled analysis of retrospective cohort studies using data from Denmark, Scotland, England, and Australia. They defined mode of delivery and assessed potential confounders using birth records and identified subsequent infant infections by record linkage to hospital discharge data from the children. Although the proportional increase in risk is relatively modest (hazard ratio 1.10, 95% confidence interval 1.09 to 1.12), as cesarean delivery is common, even this modest association leads to an attributable fractions of 1.8% to 3.2% across the different countries. However, these calculations assume causality.

One finding in favour of a causal association is the fact that it was observed with both planned and emergency cesarean delivery, given that the indications for each are very different. However, it is still possible that an unmeasured confounder explains this observation, and the relatively modest hazard ratios are consistent with this. The most common indication for emergency cesarean in first pregnancies is poor progress in labour [8]. Repeat cesarean section, both planned and emergency, accounts for a large proportion of cesarean sections in parous women [8]. Hence, it is plausible that a single unmeasured confounder, associated with both poor progress in labour and the risk of infection in the offspring, could explain the findings. However, the fact that the association was observed across all 4 countries strengthens the argument for causality. It is interesting that the association was observed across a wide range of age windows, from 0 to 3 months through to 2 to 5 years, and involved infection of multiple organ systems. The consistency of association could be interpreted as supporting causation. However, the same pattern could also be interpreted as a lack of specificity. A causal association requires an underlying mechanism, and it is important to consider what type of mechanism might explain a modest increase in risk, but one which is present across a wide range of ages and across multiple organs.

An argument in favour of a causal association is the fact that cesarean section represents—literally—an unnatural form of birth. Labour and vaginal delivery are associated with multiple stimuli for the infant, including physical, hormonal, and microbial. Extrauterine life is characterised by colonisation of the infant by the various site-specific microbiomes. Passage through the genital tract exposes the fetus to the mother’s anogenital microbiome and is thought to have an important role in the genesis of the infant’s commensal microbiota. The past 20 years have seen a massive expansion in our understanding of the importance of the various organ-specific microbiomes in the determination of health and disease. Given that mammalian physiology has, for more than 100 million years, involved this exposure of the fetus during transition from the sterile womb to the microbially diverse world, it seems plausible that entering the world through an aseptic opening could have significant effects on the infant. The area is highly controversial. Some authors have applied molecular tests to intrauterine tissues and concluded that the fetus may be colonised before birth [9]. Others have argued that these signals are artefacts, including contamination of laboratory reagents with DNA from environmental bacteria and contamination of tissues by bacteria from the mother’s genital tract during birth [10]. Some have observed prolonged alteration in the fetal intestinal microbiome in relation to cesarean delivery [11], while others have not [12]. However, there is direct experimental evidence in animal models to indicate that cesarean section can lead to altered immune responses through effects on intestinal colonisation [13], and this a candidate mechanism to explain the observations described by Miller and colleagues.

Key points for women considering this evidence in their decision-making around mode of delivery could include the following. First, human beings have evolved giving birth vaginally. As we will never have perfect information about the balance of risks and benefits, some prioritisation of what is physiological is scientifically reasonable. Second, in relation to the association documented by Miller and colleagues, the absolute risk difference is relatively small. Moreover, it is uncertain whether the actual decision about mode of delivery is causally associated with this outcome. Finally, the individual woman’s choice is not to be delivered by cesarean section or to have a vaginal birth. Rather, the alternative to elective cesarean section is to attempt vaginal birth with the possible outcomes of success or emergency cesarean delivery. For a minority of women with a high prior risk of emergency cesarean, a planned procedure may be associated with lower risks [14]. However, among the majority of women who have a high probability of vaginal delivery, the balance of risks and benefits will favour aiming for a vaginal birth. But calculating the balance of risks and benefits requires knowing both as fully as possible. It is biologically plausible that mode of delivery could have lifelong effects on the mother and baby, and studies such as this one are crucial for women to make fully informed decisions.
